# Randomized Controlled Trial of Doula-Home-Visiting Services: Impact on Maternal and Infant Health

**DOI:** 10.1007/s10995-018-2537-7

**Published:** 2018-05-31

**Authors:** Sydney L. Hans, Renee C. Edwards, Yudong Zhang

**Affiliations:** School of Social Service Administration, University of Chicago, 969 E 60th Street, Chicago, IL 60637 USA

**Keywords:** Doula, Home visiting, Breastfeeding, Safe sleep

## Abstract

*Introduction* Although home-visiting programs typically engage families during pregnancy, few studies have examined maternal and child health outcomes during the antenatal and newborn period and fewer have demonstrated intervention impacts. Illinois has developed an innovative model in which programs utilizing evidence-based home-visiting models incorporate community doulas who focus on childbirth education, breastfeeding, pregnancy health, and newborn care. This randomized controlled trial (RCT) examines the impact of doula-home-visiting on birth outcomes, postpartum maternal and infant health, and newborn care practices. *Methods* 312 young (*M* = 18.4 years), pregnant women across four communities were randomly assigned to receive doula-home-visiting services or case management. Women were African American (45%), Latina (38%), white (8%), and multiracial/other (9%). They were interviewed during pregnancy and at 3-weeks and 3-months postpartum. *Results* Intervention-group mothers were more likely to attend childbirth-preparation classes (50 vs. 10%, OR = 9.82, *p* < .01), but there were no differences on Caesarean delivery, birthweight, prematurity, or postpartum depression. Intervention-group mothers were less likely to use epidural/pain medication during labor (72 vs. 83%; OR = 0.49, *p* < .01) and more likely to initiate breastfeeding (81 vs. 74%; OR = 1.72, *p* < .05), although the breastfeeding impact was not sustained over time. Intervention-group mothers were more likely to put infants on their backs to sleep (70 vs. 61%; OR = 1.64, *p* < .05) and utilize car-seats at three weeks (97 vs. 93%; OR = 3.16, *p* < .05). *Conclusions for practices* The doula-home-visiting intervention was associated with positive infant-care behaviors. Since few evidence-based home-visiting programs have shown health impacts in the postpartum months after birth, incorporating doula services may confer additional health benefits to families.

## Significance

*What’s Known on This Subject* Research has shown that home-visiting programs have positive impacts in varied domains of parent and child functioning. However, few studies have examined maternal and child health at birth and during the newborn period.

*What This Study Adds* This study, evaluating a home-visiting model that incorporates community doulas into the intervention team, demonstrates improvements in childbirth preparation, breastfeeding initiation, safe sleep practices, and early car-seat use. The intervention was associated with less use of pharmacologic pain control during labor, but not with other indicators of mother and newborn health at birth or improvements in maternal depression.

## Introduction

### Home Visiting and Maternal Child Health

Growing evidence shows that childhood home-visiting programs for socially and economically vulnerable families can have impacts in multiple areas, including maternal and child health, parenting, child development, and family economic self-sufficiency (Paulsell et al. 2010). When federal support for home visiting was dramatically increased in 2010 through the Maternal Infant Early Childhood Home Visiting (MIECHV) program (Thompson et al. 2011), the legislation set expectations that program should have impacts across multiple domains, including “improved maternal and newborn health” (“Patient Protection and Affordable Care Act”). Although MIECHV legislation did not prioritize specific maternal and newborn health outcomes, the U.S. Department of Health and Human Services’ national health blueprint, Healthy People 2020 (Office of Disease Prevention and Health Promotion 2014), identifies such priorities: mother health at birth and postpartum (including attendance at childbirth preparation classes, reduction in Caesarean deliveries, reduction in maternal postnatal medical complications, and reduced postpartum depression), infant morbidity and mortality (including reduction in infant deaths, low birthweight and preterm birth), and infant care (including increased breastfeeding and increased proportion of infants put to sleep on their backs).

The United States lags behind other developed nations with respect to infant mortality and low birthweight (MacDorman et al. 2014; Wardlaw 2004), and there are disparities in newborn and pregnancy outcomes related to maternal age, poverty and race (Bryant et al. 2010; Martin et al. 2017; Nagahawatte and Goldenberg 2008). Despite evidence that breastfeeding has advantages for mother and child health (Stuebe 2009), breastfeeding rates remain low in the US among young, low-income and African-American women (McDowell et al. 2008). Additionally, although the American Academy of Pediatrics (Task Force on Sudden Infant Death Syndrome 2016) recommends that infants be placed in supine sleep positions in their own beds in order to reduce the risk of sleep-related infant deaths, infants born to young, low-income mothers have a relatively high risk for prone placement and for co-sleeping (Colson et al. 2009; Caraballo et al. 2016).

Despite many studies on infant and early childhood home visiting, few reports document impacts on maternal and newborn health or newborn care practices. Only a few home-visiting studies have examined maternal depression during the first postpartum months, and none have found program impacts reducing symptoms (e.g., Barlow et al. 2013; Carta et al. 2013). A few studies have shown impacts on preventing low birthweight and/or preterm birth (Lee et al. 2009; Williams et al. 2017), but others have not (e.g., Kitzman et al. 1997; Olds et al. 1986). Most studies have not examined newborn health. Some home-visiting studies have reported impacts on early breastfeeding (Kitzman et al. 1997; Wen et al. 2011), but most have not found impacts (Green et al. 2014; Kemp et al. 2013; Mitchell-Herzfeld et al. 2005).

### Community Doulas

Twenty years ago, early childhood advocates in Illinois were concerned about home-visiting programs having limited impact on maternal and newborn health outcomes. A partnership between the Irving Harris Foundation, HealthConnect One and the Ounce of Prevention Fund developed a model where doulas were integrated into home-visiting programs in order to enhance the quality of health-related services during pregnancy and the postnatal period (Glink 1998, 1999).

In the “community doula” model that resulted, doulas are community health workers who have training in pregnancy health, childbirth preparation, labor support, lactation counseling, and newborn care. They serve as specialized home visitors, providing home-based education and support during the last half of pregnancy and for 6 weeks postpartum. Doulas accompany laboring women to the hospital to provide comfort measures and emotional support and to offer postpartum help around breastfeeding and bonding.

The rationale for including doulas within a home-based model drew from strong meta-analytic evidence that doula labor support is associated with improved health outcomes, including fewer Caesarean deliveries, decreased use of analgesia/anesthesia, shorter labors, and higher Apgar scores (Hodnett et al. 2013). One RCT examining the impact of a community doula model in which doulas provided home visits in addition to labor support found increases in breastfeeding initiation among young, low-income mothers (Edwards et al. 2013).

The goal of this RCT is to examine whether young, low-income families receiving doula-home-visiting services, compared to families receiving lower-intensity case-management services, have improved maternal and child health outcomes during the period between birth and 3 months of age.

## Methods

### Study Sites, Enrollment, Randomization and Follow-Up Procedures

Study recruitment took place between 2011 and 2015. Partners in the RCT were four agencies offering doula-home-visiting programs to young mothers in high-poverty Illinois communities. Two programs were located in a large city, and two in smaller urban areas. One served an African-American population, one served a Latinx population, and two served mixed-ethnic populations. Programs serving Latinx populations provided services in English and Spanish. Each of the programs already was implementing an evidence-based home visiting model (see overview of evidence in Paulsell et al. 2010), either Healthy Families America (HFA) (“Healthy Families America” 2015) or Parents as Teachers (PAT) (“Parents as Teachers” 2018). Programs were from a network of state-funded home-visiting programs and not demonstration programs for research purposes only.

Programs received information about young pregnant women from their usual referral networks—public health departments, WIC programs, health clinics, and schools. Program staff contacted women to determine eligibility and explain the program and research study. Women were told that the only way to participate in the doula-home-visiting program was to participate in the study. If they were not interested in the research, they received contact information for other community programs providing services for pregnant women, including case management, home visiting, and parenting programs in hospitals and health clinics. To be eligible for the study, women needed to be under 26, less than 34 weeks gestation, living in the program geographic catchment area, planning to remain the area, and meeting sociodemographic risk criteria used by the HFA or PAT models. Out of ethical concerns, pregnant women who were under 14, involved with the child welfare or juvenile justice systems, or had significant cognitive impairments were excluded from the study and offered home-visiting services.

After screening, the research team scheduled a baseline session with mothers that included a written-informed-consent procedure and a 2-h structured interview. At the end of this session, the interviewer opened a sealed opaque envelope that showed whether the participant was assigned to doula-home-visiting services (intervention condition) or case management (control condition). These envelopes had been prepared by the principal investigator before the beginning of the study. Randomization tables were created separately for each community.

At 37-weeks of pregnancy, 3-weeks postpartum and 3-months postpartum, mothers were re-interviewed. Families received modest monetary compensation at each session and a baby book and toy at each postpartum session. All study procedures were approved by the Institutional Review Board at The University of Chicago, and the study is registered with clinicaltrials.gov [identifier NCT01947244].

### Description of Group Conditions

#### Doula-Home-Visiting Intervention

After randomization to the intervention group, doula-home-visiting programs assigned families a home visitor (also called a Family Support Worker or Parent Educator) and a community doula. Doulas and home visitors all had deep roots in their communities. All home visitors and doulas had completed at least the foundational training required by their national models, and doulas had completed at least the basic training provided through the Ounce of Prevention Fund. During pregnancy and postpartum, mothers were visited weekly by a home visitor, doula, or both together. The doula worked with the mother more intensively during pregnancy and the first weeks postpartum, while the home visitor became the primary provider by 6 weeks postpartum.

Home visitors focused on the mother-infant relationship, child development, child safety, and educational-work planning, as well as screening to make sure that family basic needs were being met. Doulas focused on issues related to pregnancy health, childbirth preparation, breastfeeding, newborn care, postpartum health, and early bonding. Doulas sometimes accompanied mothers to prenatal and postpartum medical visits. Doulas attended births at the hospital where they provided mothers with physical comfort, emotional support, and advocacy during labor and delivery and breastfeeding counseling postpartum. Doulas also offered prenatal classes at the program sites. All programs conducted regular depression screenings and made referrals to mental health consultants.

### Case Management Control

After randomization to the control group, mothers were provided information about case management services in their communities, and case management providers were given mothers’ contact information. In some communities, mothers were referred to existing state-funded case-management providers; in other communities, social-service providers were contracted to provide case management. It was expected that mothers would have at least two meetings with case managers—one during pregnancy and one after birth. Meetings could be in families’ homes, in agency offices, or occasionally by phone. Case managers determined whether families’ basic needs with respect to health, housing, food, employment, education, and childcare were being met, and if needed, made referrals. Case managers screened to identify needs for services regarding substance misuse, depression, and domestic violence.

### Interviews

Outcomes were chosen based on Healthy People 2020 maternal and newborn health priorities and outcomes that have been reported in previous studies of doula interventions. Interviews were available in English and Spanish and administered in the mother’s preferred language. Interviewers working in Latinx communities were bilingual. Interviews were usually conducted in families’ homes.

At baseline, interviewers asked questions related to the pregnancy, health care, mental health, education and employment, and relationships with family. Baseline interview questions were used to check equivalence of the groups as randomized.

At all follow-up interviews, intervention-group mothers were asked about numbers of contacts with doulas and home visitors. All mothers were asked about childbirth preparation class attendance and any other pregnancy/parenting services.

At the 3-week postpartum interview (or 3-month interview if mother missed the earlier session), mothers reported on birth outcomes, including pain medication/epidural use during labor, vaginal versus Caesarean delivery, gestational age (GA) at delivery, infant birthweight, NICU admission, length of hospital stay, and mother and/or newborn re-hospitalizations.

At the 3-week and 3-month interviews, mothers reported on breastfeeding. Breastfeeding initiation was defined as breastfeeding at least through the hospital stay. Mothers were asked how often they used a car-seat, the positions they used when laying down their infant to sleep and where the infant slept. Mothers reported on depressive symptoms using the Center for Epidemiological Studies-Depression Scale (CES-D) (Radloff 1977), dichotomized to identify mothers with clinically significant levels of depression (≥ 16).

### Analytic Plan

First, the intervention and control groups were compared on multiple baseline maternal characteristics measured before randomization using *t*-tests and Chi square tests to check whether randomization was successful. Second, intent-to-treat logistic regression analyses were conducted to examine the impact of the doula-home-visiting intervention on outcomes measured at 37-weeks pregnancy, 3-week postpartum, and 3-months postpartum. Odds ratios, 95% confidence intervals, and one-tailed *p*-values were calculated for each outcome, using the control group as the reference group. Program site was used as a covariate in all analyses, and any baseline maternal variables that differed between the two groups were used as covariates.

## Results

### Sample Characteristics

Altogether 436 women were referred to the programs. 312 were enrolled in the sample and randomly assigned to the two conditions. Reasons families were not enrolled included inability to contact, women not wanting to participate in services or the study, women not meeting eligibility criteria, and women at high risk and referred to program services without randomization.

**Fig. 1:**
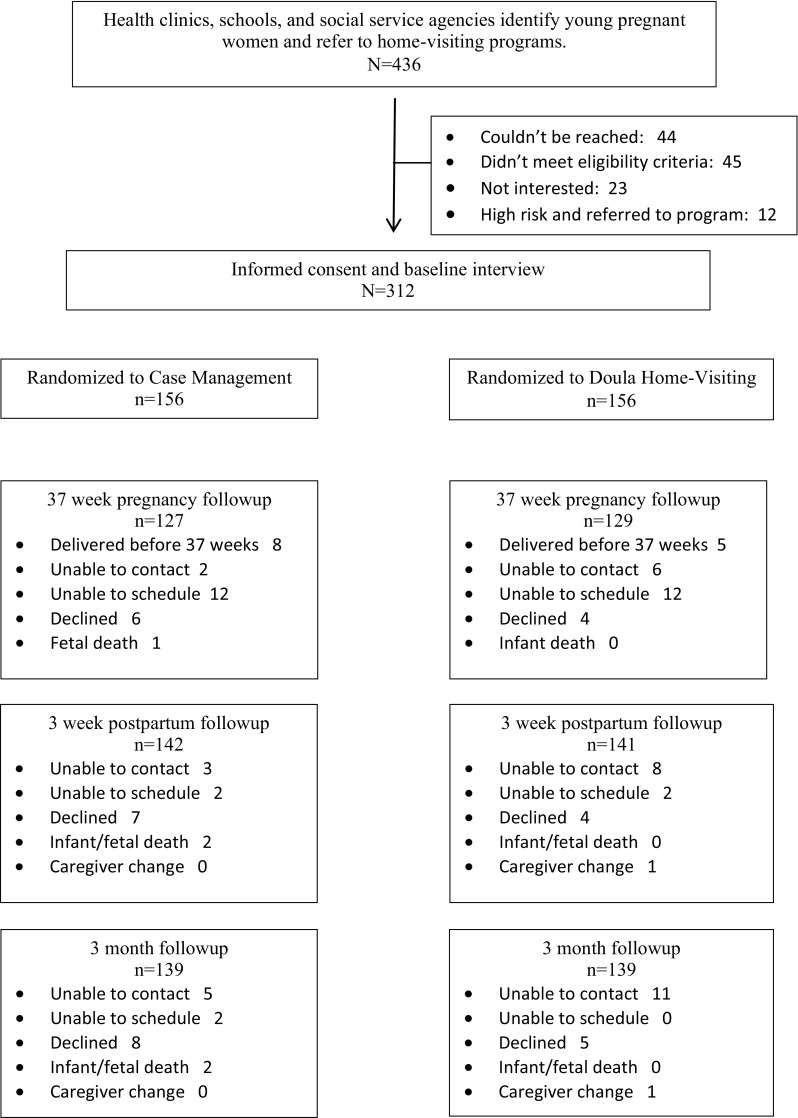
Study CONSORT diagram

Interviews were completed for 256 mothers (82%) at 37-weeks of pregnancy, 283 mothers (91%) at 3-weeks and 278 mothers (89%) at 3-months. Sample attrition was unrelated to program site, race/ethnicity, age, education, co-residence, or prenatal depressive symptoms. There were no differences in sample attrition at either follow-up interview between the intervention and control groups. Figure 1 is a CONSORT chart identifying the flow of subjects through the study.

Participants in the baseline interview were young and low-income, with almost half identifying as black/African American (45%, n = 140) and just over a third Latina/Hispanic (38%, n = 117). 11% of mothers preferred to be interviewed in Spanish. Most mothers were in their second trimester of pregnancy and expecting their first child. Over two-thirds were partnered (coupled, engaged, married) with the father of the baby (71%, n = 220). Table 1 shows that the only baseline difference between groups was that more intervention-group mothers were living with a parent figure compared to control-group mothers (77 vs. 64%, *p* < .05). Co-residence with parent figure was a control variable in all analyses.

**Table 1:** Characteristics of doula-home-visiting intervention group and control group at enrollment

		Control group	Doula/HV group
		n = 156	n = 156
Mother age in years	M (SD)	18.3 (1.6)	18.5 (2.0)
Mother years of school completed	M (SD)	10.9 (1.5)	10.9 (1.5)
Mother race/ethnicity	n (%)		
African American		72 (46.2%)	68 (43.6%)
Latina/Hispanic		56 (35.9%)	61 (39.1%)
White		13 (8.3%)	13 (8.3%)
Multiracial/other		15 (9.6%)	14 (9.0%)
Mother attends school	n (%)	78 (50.0%)	86 (55.1%)
Mother employed	n (%)	28 (18.0%)	31 (19.9%)
Mother expecting first child	n (%)	154 (98.7%)	152 (97.4%)
Baby gestational age in weeks	M (SD)	25.7 (5.9)	25.5 (6.0)
Mother has received prenatal care	n (%)	154 (98.7%)	155 (99.4%)
Mother receives public insurance (n = 305)	n (%)	138 (90.8%)	140 (91.5%)
Mother receives WIC	n (%)	137 (87.8%)	131 (84.0%)
Mother depressive symptoms (CES-D)	M (SD)	14.2 (9.2)	13.8 (8.5)
Co-residing with own mother or other parent figure^a^	n (%)	100 (64.1%)	120 (76.9%)
Co-residing with baby’s father	n (%)	48 (30.8%)	39 (25.0%)
Partnered with baby’s father	n (%)	107 (68.6%)	113 (72.4%)

^a^Chi-square test shows significant difference between intervention and control groups at *p* < .05

### Intervention Participation

Virtually all mothers (99%, n = 153) assigned to the doula-home-visiting group received at least one home visit. Among mothers interviewed at 37 weeks, the average number of doula visits prior to 37 weeks was 8.9 (SD = 6.9) and the average number of visits from a home visitor was 5.8 (SD = 4.8). Doulas were present in the hospital for 75% (n = 106) of the births. By 3-months postpartum, 131 (92%) mothers had received at least one postpartum visit from their doula and 120 (84%) had received at least one postpartum visit from their home visitor.

### Intervention Effects

#### Mother Birth and Postpartum Health

Results from logistic regression analyses using one-tailed hypothesis tests (Table 2) show that intervention-group mothers were more likely to attend a childbirth education class during pregnancy (OR 9.82, 95% CI 4.84–19.89) and less likely to use epidural or other pain medication during labor compared to control-group mothers (OR 0.47, 95% CI 0.25–0.88). The intervention was not associated with Caesarean deliveries, mother re-hospitalizations, or mother postpartum depressive symptoms.

**Table 2:** Intervention impacts on maternal health, newborn health, and newborn care outcomes

	Control group n (%)	Doula/HV group n (%)	OR [95% CI]^a^	*p*-value (1-tailed)
Mother birth and postpartum health				
Entered labor having attended childbirth preparation class (n = 255)	12 (9.5%)	64 (50.0%)	9.82 [4.84, 19.89]	0.00
C-section birth (n = 286)	31 (21.5%)	33 (23.2%)	1.04 [0.59, 1.84]	ns
Epidural/pain medication use during labor (n = 268)	114 (83.2%)	94 (71.76%)	0.47 [0.25, 0.88]	0.01
Mother re-hospitalized within 3 weeks (n = 286)	3 (2.1%)	4 (2.8%)	1.53 [0.33, 7.21]	ns
3 week high depressive symptoms (n = 282)	31 (21.8%)	31 (22.1%)	0.96 [0.53, 1.71]	ns
3 month high depressive symptoms (n = 277)	21 (15.1%)	18 (13.0%)	0.95 [0.47, 1.91]	0.45
Infant morbidity and mortality				
Fetal/newborn death (n = 286)	2^b^ (1.3%)	0 (0.0%)	–	–
Preterm birth (GA < 37 weeks; n = 285)	12 (8.2%)	10 (6.7%)	0.57 [0.22, 1.46]	0.18
Low birthweight (n = 285)	13 (9.0%)	9 (6.4%)	0.64 [0.26, 1.59]	0.17
NICU admission (n = 286)	23 (16.0%)	21 (14.8%)	0.87 [0.45, 1.68]	0.34
Hospital stay ≥ 4 days (n = 286)	28 (19.4%)	25 (17.6%)	0.89 [0.48, 1.63]	0.35
Has pediatrician at 3 weeks (n = 282)	139 (97.9%)	138 (98.6%)	1.56 [0.25, 9.65]	0.32
Has pediatric checkup by 3 months of age (n = 278)	139 (100.0%)	139 (100.0%)	–	–
Infant re-hospitalized within 3 weeks^c^ (n = 284)	5 (3.6%)	3 (1.4%)	0.45 [0.08, 2.48]	0.18
Newborn care practices				
Breastfeeding initiation (n = 287)	107 (74.3%)	116 (81.1%)	1.67 [0.91, 3.03]	0.05
Breastfeeding at 3 months (n = 278)	31 (21.8%)	24 (16.9%)	0.85 [0.45, 1.60]	ns
Always puts infant on back to sleep at 3 weeks (n = 282)	86 (60.6%)	98 (70.0%)	1.64 [0.97, 2.77]	0.03
Always puts infant on back to sleep at 3 months (n = 277)	83 (60.1%)	92 (66.2%)	1.34 [0.80, 2.23]	0.13
Infant sleeps in own bed at 3 weeks (n = 282)	63 (44.4%)	74 (52.9%)	1.44 [0.89, 2.34]	0.07
Infant sleeps in own bed at 3 months (n = 277)	67 (48.6%)	71 (51.1%)	1.19 [0.72, 1.95]	0.25
Always uses car seat at 3 weeks (n = 281)	132 (93.0%)	135 (97.1%)	3.67 [1.06, 12.70]	0.02
Always uses car seat at 3 months (n = 277)	126 (91.3%)	130 (93.5%)	1.28 [0.51, 3.20]	0.30

^a^Logistic regression analyses control for co-residence with parent figure at baseline and program site

^b^A third infant from the control group died before age 4 months of age

^c^Two infants were in the hospital continuously from birth through 3 weeks of age so were excluded from analyses on re-hospitalizations

### Infant Mortality and Morbidity

The intervention was not associated with preterm births (GA < 37 weeks), low birthweight, NICU admission, length of newborn hospital stay, re-hospitalization of infants, having a pediatrician or pediatric clinic at 3 weeks, or having a well-baby check up by 3 months. Almost all families in both groups reported having a pediatrician for their infants (98%), and all mothers reported taking their infant in for at least one well-baby check up by 3 months of age.

### Newborn Care Practices

Mothers in the intervention group were more likely to initiate breastfeeding in the hospital (OR 1.67, 95% CI 0.91–3.03). At 3 weeks, mothers in the intervention group were more likely to always place their infants on their backs for sleeping (OR 1.64, 95% CI 0.97–2.77) and to always put their infants in a car seat when traveling by car (OR 3.67, 95% CI 1.06–12.70). There was a non-statistically-significant trend for infants in the intervention group to have their own beds (OR 1.44, 95% CI 0.89–2.34, *p* = .07). There were no group differences on breastfeeding, sleeping or car seat use at 3 months.

## Discussion

Although most early-childhood home-visiting programs begin working with families during pregnancy or soon after birth, relatively few evaluations have examined maternal and child health outcomes at birth or during the newborn period. The doula-home-visiting model, in which a community doula partners with a home visitor during pregnancy and through 6-weeks postpartum, provides greater emphasis on pregnancy health, childbirth, breastfeeding, and newborn health than most other home-visiting models, and additionally, offers hospital-based support during childbirth and agency-based childbirth preparation classes. This RCT shows that the doula-home-visiting intervention has impacts on childbirth preparation, epidural/pain medication use during labor, breastfeeding, and safe newborn-care practices.

Mothers receiving the intervention were more likely to have attended a childbirth preparation class. Although virtually all mothers in the sample had opportunities to attend childbirth classes through prenatal clinics and hospitals, few control-group women took advantage of such opportunities. Half of the women in the intervention group participated in such classes either at clinics and hospitals or through weekly classes offered by their home-visiting programs. Moreover, all mothers who were visited by a doula also received individualized childbirth education at home. Perhaps as a result of this preparation and the presence of the doula during labor, mothers in the intervention group were less likely to use pharmacologic pain relief during labor, a finding similar to other studies of doula labor support (Hodnett et al. 2013). However, as with the few other home-visiting studies examining birth outcomes, there were no intervention impacts on Caesarean deliveries, low birthweight, or preterm birth. Although other studies of labor-only doulas have found reductions of Caesarean rates (Hodnett et al. 2013), most of these studies limited samples to obstetrically low-risk mothers whose labors began spontaneously. The present sample of young, low-income mothers was likely more medically complex.

Mothers in the intervention were more likely to initiate breastfeeding, consistent with previous research on community doulas (Edwards et al. 2013). Few other home-visiting studies have found impacts on breastfeeding. Doulas, by offering skilled lactation counseling throughout pregnancy in mothers’ homes and postpartum in the hospital, increase breastfeeding initiation, even among populations that have traditionally low breastfeeding rates. However, the intervention impact on breastfeeding was not sustained, and only about 20% of mothers were breastfeeding at 3 months. Research is needed to understand why many mothers initiated breastfeeding but discontinued quickly postpartum (e.g., Rozga et al. 2015) and what strategies might be effective for supporting young mothers during that critical time. Nevertheless, even brief periods of breastfeeding may have health benefits to infants by way of colostrum (e.g., Bardanzellu et al. 2017).

The doula-home-visiting intervention did not show impacts on postpartum maternal depressive symptoms, consistent with findings from most other home-visiting evaluations. Postpartum depression is powerfully influenced by a complex set of biological factors, chronic stress, trauma history, and instability in relationships with infants’ fathers and have been challenging to prevent (Edwards et al. 2012; Grote et al. 2011). A systematic review found evidence that home-based services have the potential to be effective in preventing postpartum depression, but to date evidence is limited to intensive interventions delivered by professionals (Dennis and Dowswell 2013).

Mothers in the intervention were more likely to always place their newborns on their backs to sleep and always use a car-seat. Few previous home-visiting studies have looked at early infant safety practices. Although the present study does not address the manner in which mothers received these safety messages, previous research suggests that low-income mothers may reject infant sleep recommendations, for example, because of distrust of health professionals, reliance on advice from family members, and concern for infant comfort (Colson et al. 2005). Doulas have many opportunities during prenatal visits and through their intimate care during labor to become trusted advisors to young mothers. By being present in the hospital and the home during the earliest weeks when mothers first establish sleep practice, doulas may have unique opportunities to explain to mothers and other family members the benefits of safe sleep practices and to offer mothers strategies for soothing babies who seem uncomfortable on their backs.

Notably, although doula-home-visiting impacts on newborn care practices were found in the first weeks postpartum, group differences diminished by 3 months. It may be that over time families chose infant feeding or sleeping practices they felt were most effective for their family circumstances or infant preferences. It may be that as home visitors took over intervention work from the doulas, the focus of the work shifted from feeding and sleep practices to other important areas such as responsive parenting, child development, and mother’s personal development.

Finally, although the present study has many methodological strengths—a randomized design implemented within programs taken to scale in real agency settings, it also has limitations. The sample drew from only four programs in a single state and excluded adolescents at the most extreme levels of risk. Because data in this paper were provided through mother report and not administrative records, reliable information on important medical procedures and outcomes during labor, such as qualifications of health providers and Apgar scores, were unavailable. Because each mother in the intervention group was offered services from a doula and home visitor team, the independent contribution of the two different providers could not be determined. The sample was underpowered to detect important, but relatively rare maternal and child health problems, particularly infant mortality. Nevertheless, the study identified impacts on important maternal and newborn health outcomes that have rarely or never been found in other evaluations of home-visiting models. Future research should focus on a broader set of health outcomes, including outcomes documented through administrative records, and examine the processes through which doulas convey health information, contrasting their role to home visitors who are not doulas.

Consistent with an already strong evidence base regarding birth doula interventions and a smaller body of work on community doulas, the present study shows improved maternal and child health when mothers have access to doula services through community-based home-visiting programs. However, there are presently funding barriers to increasing low-income women’s access to doula services. Simple steps to improving access would be for states to recognize community doula services as evidence-based interventions eligible for existing home-visiting funding, as has been the case in Illinois, and also to develop state certification processes and other mechanisms for using Medicaid funds to reimburse for doula services, as has happened in Oregon and Minnesota (Gay 2016; Kozhimannil et al. 2014).
