# Correction to: Randomized Controlled Trial of Doula-Home-Visiting Services: Impact on Maternal and Infant Health

**DOI:** 10.1007/s10995-018-2626-7

**Published:** 2018-08-20

**Authors:** Sydney L. Hans, Renee C. Edwards, Yudong Zhang

**Affiliations:** School of Social Service Administration, University of Chicago, 969 E 60th Street, Chicago, IL 60637 USA

## Correction to: Maternal and Child Health Journal 10.1007/s10995-018-2537-7

The article “Randomized Controlled Trial of Doula-Home-Visiting Services: Impact on Maternal and Infant Health”, written by Sydney L. Hans, Renee C. Edwards and Yudong Zhang, was originally published electronically on the publisher’s internet portal (currently SpringerLink) on 31 May 2018 without open access. With the author(s)’ decision to opt for Open Choice the copyright of the article changed on 18 July 2018 to © The Author(s) 2018 and the article is forthwith distributed under the terms of the Creative Commons Attribution 4.0 International License (http://creativecommons.org/licenses/by/4.0/), which permits use, duplication, adaptation, distribution and reproduction in any medium or format, as long as you give appropriate credit to the original author(s) and the source, provide a link to the Creative Commons license and indicate if changes were made.

The original article has been corrected.

